# Modified Citrus Pectin Treatment in Non-Metastatic Biochemically Relapsed Prostate Cancer: Long-Term Results of a Prospective Phase II Study

**DOI:** 10.3390/nu15163533

**Published:** 2023-08-11

**Authors:** Daniel Keizman, Moshe Frenkel, Avivit Peer, Eli Rosenbaum, David Sarid, Ilan Leibovitch, Roy Mano, Ofer Yossepowitch, Ido Wolf, Ravit Geva, David Margel, Keren Rouvinov, Anat Stern, Hadas Dresler, Igal Kushnir, Isaac Eliaz

**Affiliations:** 1 Department of Oncology, Tel Aviv Sourasky Medical Center, School of Medicine, Tel Aviv University, Tel Aviv 69978, Israel; davids@tlvmc.gov.il (D.S.); idow@tlvmc.gov.il (I.W.); ravitg@tlvmc.gov.il (R.G.); 2 Department of Oncology, Rambam Medical Center, Haifa 3200003, Israel; frenkelm@netvision.net.il (M.F.); a_peer@rambam.health.gov.il (A.P.); 3 Department of Oncology, Rabin Medical Center, Petah Tikva 4941492, Israel; elimiriam1@yahoo.com; 4 Department of Urology, Meir Medical Center, Kfar Saba 4428164, Israel; ilan.leibo@gmail.com; 5 Department of Urology, Tel-Aviv Sourasky Medical Center, Tel Aviv 69978, Israel; roymano78@gmail.com (R.M.); ofery@tlvmc.gov.il (O.Y.); 6 Department of Urology, Rabin Medical Center, Petah Tikva 4941492, Israel; sdmargel@gmail.com; 7 Department of Oncology, Soroka Medical Center, Beer Sheva 8410501, Israel; kerenruv@gmail.com; 8 Amitabha Medical Clinic and Healing Center, Santa Rosa, CA 95403, USA; astern@econugenics.com (A.S.); isaac.eliaz@gmail.com (I.E.); 9 Department of Oncology, Shaare Zedek Medical Center, Jerusalem 9124001, Israel; hadasdr@gmail.com; 10 Department of Oncology, Meir Medical Center, Sackler School of Medicine, Tel Aviv University, Tel Aviv 69978, Israel; igi_ku@yahoo.com

**Keywords:** modified citrus pectin, non-metastatic biochemically relapsed prostate cancer

## Abstract

The optimal therapy for patients with non-metastatic biochemically relapsed prostate cancer (BRPC-M0) after local therapy is elusive. Thus, the evaluation of new non-toxic compounds in BRPC-M0 patients is warranted. PectaSol^®^-Modified citrus pectin (P-MCP) is a food supplement categorized as GRAS (Generally Recognized As Safe) by the FDA. It is a competitive inhibitor of the galectin-3 protein, which is involved in cancer pathogenesis. In an early report of the present phase 2 study, P-MCP treatment for 6 months led to prostate-specific antigen doubling time (PSADT) improvement in 75% of patients with BRPC-M0. Herein, we report the second long-term treatment phase of an additional 12 months of P-MCP therapy (4.8 g × 3/day orally) in patients without disease progression after the initial 6 months of therapy. Of the 46 patients that entered the second treatment phase, 7 patients withdrew consent and decided to continue therapy out of pocket, and 39 initiated the second treatment phase. After a total of 18 months of P-MCP treatment, 85% (*n* = 33) had a durable long-term response, with 62% (*n* = 24) showing decreased/stable PSA, 90% (*n* = 35) PSADT improvement, and all with negative scans. No patient had grade 3/4 toxicity. In conclusion, P-MCP may have long-term durable efficacy and is safe in BRPC-M0.

## 1. Introduction

Globally, prostate cancer is the second most common solid tumor [1] and cause of cancer-related death in men from Westernized countries [2]. Although its etiology needs further elucidation, risk factors for prostate cancer development include age, family history, lifestyle, and hereditary syndromes [3]. With an age-standardized incidence rate of 31 per 100,000 [1] and approximately 1.5 million new cases reported in 2020 [1,4], prostate cancer incidence is increasing worldwide [4,5] and presents a significant economic burden [6]. However, with the rapid evolution of treatment options [7], and changes in screening protocols [8], prostate cancer-related mortality patterns have stabilized [5]. Primary treatment options for localized prostate cancer include radical prostatectomy, radiation therapy, and androgen deprivation therapy (ADT) for advanced cases [9]. Within 10 years of treatment, 20–50% of patients will experience a biochemical relapse, with rising PSA and negative scans (BRPC-M0) [9,10,11,12,13]. In BRPC-M0 state, patients may remain asymptomatic and free of clinical evidence of disease for many years [14], and the PSA doubling time (PSADT) is a significant prognostic factor for development of future metastases and mortality [10,13,15,16,17]. The optimal therapy of patients with BRPC-MO is elusive [10,11,17,18,19,20,21,22] Androgen Deprivation Therapy (ADT) in this setting is associated with an uncertain survival benefit, may negatively impact quality of life, and increase comorbidities such as cardiovascular [10,14,23,24,25].

Thus, there is a need to evaluate new non-toxic therapies for BRPC-M0 [2,10,11,26,27,28,29,30,31,32,33,34,35,36,37,38,39,40]. Pectins comprise carbohydrate-soluble fiber found in plant cell walls and are indigestible to humans in their unmodified form [39]. Modified citrus pectin (MCP), with shorter polysaccharide units, are water soluble [39] and have demonstrated significant anticancer activity [38,39,41,42], potentially by disrupting tumor-promoting signaling by binding galactose-containing side chains to the carbohydrate recognition domains of galectins [38,43,44]. Galectins comprise an evolutionarily conserved family of endogenous glycan-binding proteins and they play multifunctional roles in tumor progression [38] through modulating and recalibrating inter- and intra-cellular signaling [45]. Galectins affect cellular responses, including cell aggregation, growth, differentiation, apoptosis, and proliferation [38,45,46], and may promote immune evasion of cancer cells [47]. Galectins, including Galectin1 (GAL1) and GAL3, have therefore been explored as potentially effective therapeutic targets for cancer patients [38,48,49].

PectaSol^®^-Modified Citrus Pectin (P-MCP; EcoNugenics Inc., Santa Rosa, CA, USA) is a commercially available polysaccharide and is a Galectin-3 (Gal-3) inhibitor, binding to the Galectin-3 carbohydrate recognition domain [38,43]. Derived from the pith of citrus fruit peels, and classified by the US-FDA as generally regarded as safe (GRAS), data suggest that P-MCP is associated with cytotoxicity and inhibition of cancer cells [39,50], including prostate cancer cells [30,38,41,42].

In clinical trials evaluating new compounds in BRPC-MO, common endpoints include PSA dynamics as reflected by the PSADT [17,21,51,52] and are used as surrogate endpoints, both as predictive and as stratification factors for clinical disease progression [17,53]. Prolongation of PSADT may be an early signal of efficacy of active compounds in clinical trials [24,26,54].

In previous clinical trials in BRPC-MO, P-MCP therapy was associated with a positive effect on prostate-specific antigen (PSA) dynamics, including a decrease in PSA level and lengthening of PSADT in a significant proportion of patients, [30,41]. A prolongation of PSADT was shown with P-MCP therapy for 6 [30] and 12 [41] months. Herein, we report the efficacy with an extended total treatment period of 18 months, using the cohort reported previously by Keizman et al. (Nutrients 2021) [30], for which an early benefit was reported after 6 months of P-MCP therapy.

## 2. Methods and Materials

**Figure 1:**
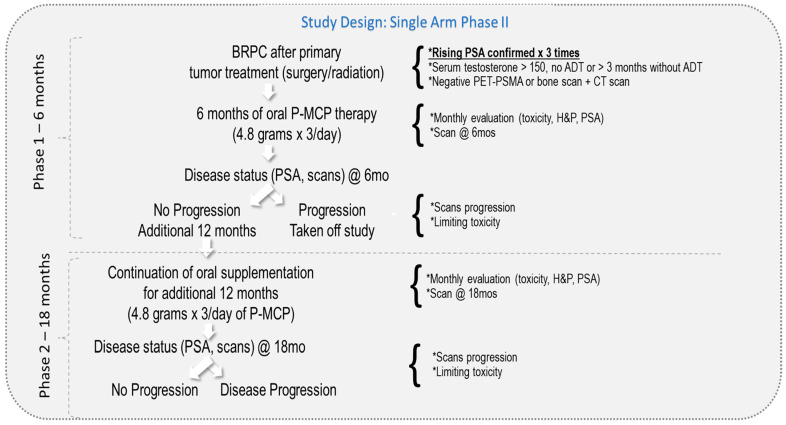
Study design.

Study design: The eligibility criteria are described in our previous publication [30]. Briefly, patients with BRPC-M0, rising PSA post-primary therapy (surgery and/or radiation), and negative scans were included. All patients had a normal level of serum testosterone > 150 ng/mL and Eastern Cooperative Oncology Group (ECOG) performance status ≤ 2 at study entry. All participating patients signed an Institutional Review Board (IRB)-approved consent form. Study participants were recruited between 2013 and 2019 from 5 medical centers in Israel (Meir, Rabin, Rambam, Soroka, and Tel-Aviv Sourasky). Patients were given 4.8 g of P-MCP to take orally 3 times per day for the duration of the study. The P-MCP was provided by PectaSol-C^®^, EcoNugenics, Santa Rosa, CA, USA, in packs of 270 capsules. Patients without evidence of disease progression or dose-limiting toxicity after 6 months of therapy (*n* = 46) entered the second long-term phase of the study and were given an additional 12 months of treatment. A total of 39 patients completed the full 18 months of treatment. The study design is described in Figure 1.

Evaluation of disease status: Patients underwent monthly visits for toxicities, physical examinations, and serum PSA, with baseline measurements taken prior to starting the P-MCP treatment protocol. A positron emission tomography (PET)—prostate-specific membrane antigen (PSMA) scan was conducted after 6 and 18 months in patients without clinical or PSA progression, or earlier upon clinical or PSA progression. The primary efficacy endpoint was the rate of patients without PSA progression (defined as an increase of ≥ 25% from baseline) and/or patients with improvement (lengthening) of PSADT versus baseline. The post-baseline PSADT was calculated using baseline PSA measurements obtained at the start of the study and every month during treatment. Secondary endpoints included the rate of patients without radiologic progression and toxicity, and with treatment benefits according to the PSADT risk grouping (e.g., poor < 3 months, intermediate 3–8.99 months, and good ≥9 months) [30].

Duration of treatment: Treatment as per the protocol continued for 12 additional months or until biochemical or clinical disease progression or dose-limiting toxicity.

Disease progression was defined as biochemical progression without PSADT prolongation and/or new radiological evidence of metastases.

Safety evaluation of toxicity: Toxicity was defined according to the National Cancer Institute (NCI) Common Toxicity Criteria, with treatments terminated at grades 3/4 and patients monitored weekly until ≤grade 1 before restarting therapy. Treatment would be discontinued upon the recurrence of a same grade 3/4 event and for any toxicity requiring longer than 4 weeks to recover to ≤grade 1.

Statistical analysis: Comparisons between pre- and post-treatment endpoint parameters, and within groups were analyzed using the Wilcoxon Signed Rank test for abnormally distributed data, or the two-tailed Student *t*-test for normally distributed data, with results reported as number, percentage, mean or median, and standard deviation (SD). A *p*-value ≤ 0.05 was considered statistically significant.

Regulatory Considerations: The research was conducted in accordance with the approval by the IRB committee of our institution. The study was registered at ClinicalTrials.gov (accessed on 10 September 2012) (NCT01681823).

## 3. Results

Patients: Of the 46 patients that displayed a benefit from the initial 6 months of therapy (in terms of stabilization/decrease of PSA, and/or improvement of PSADT, and with negative scans) and that were eligible for the second phase of an additional 12 months of therapy (to create a total of 18 months of treatment), 7 patients withdrew consent during the first month of the additional year of therapy and chose to continue the effective therapy independently, out of pocket, due to the travel distance to monthly medical visits. Thus, 39 patients were treated as per the protocol for a total of 18 months of treatment. Patient pre-treatment characteristics are summarized in Table 1.

**Figure 2:**
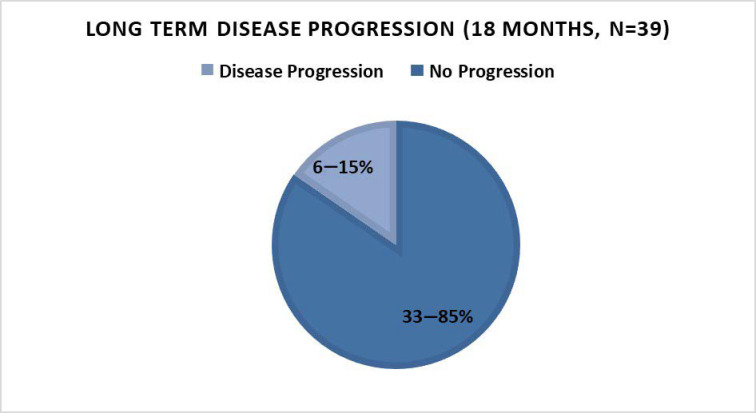
Disease progression status after 18 months of PectaSol^®^-Modified Citrus Pectin (P-MCP) therapy.

Long-term outcome as determined by PSA level, PSADT, and disease progression: Out of the 39 patients that entered the second phase of the study (the additional 12 months of therapy, creating a total of 18 months), 85% (*n* = 33) demonstrated a decreased or stable PSA (Figure 2) and/or improvement of PSADT (54%, *n* = 21), with negative scans (90%, *n* = 35). Median PSADT improved significantly versus baseline (*p* = 0.003), from a median pre-treatment PSADT of 10.3 (median range = 1.4–54.6) months to a median post-treatment PSADT of 43.5 (median range = 3.5–981.0) months (Table 1 and Table 2).

**Figure 3:**
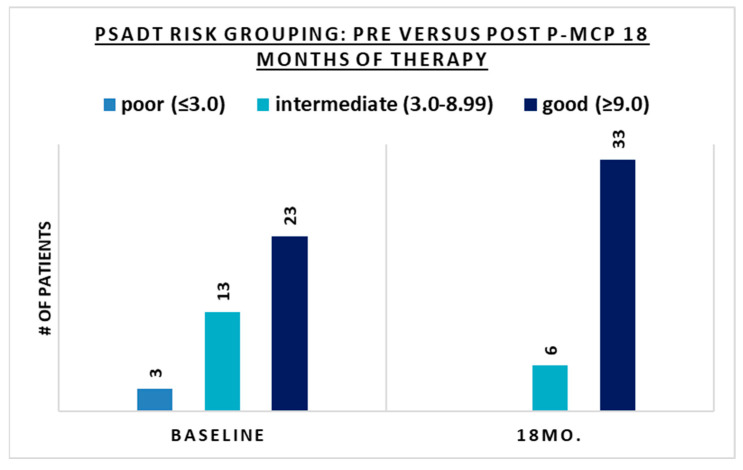
The number of patients in each PSADT risk group at baseline and after 18 months of PectaSol^®^-Modified Citrus Pectin (P-MCP) therapy.

The benefits of 18 months of therapy in terms of PSA stabilization or decrease and/or PSADT lengthening were seen in all PSADT risk groups (Table 2). There was also an improvement in PSADT risk grouping after 18 months of treatment (Table 2 and Figure 3). After 18 months of P-MCP therapy, all patients with a pre-treatment PSADT risk grouping of poor (<3 months) improved their PSADT to an intermediate–good risk, and most patients (77%) with a pre-treatment risk PSADT of intermediate improved their PSADT risk to good (Table 2 and Figure 3). In patients with a good risk pre-treatment PSADT, 91% retained their risk grouping, 87% of whom had an improved PSADT. After 18 months of therapy, no patients remained in the poor risk PSADT group, and the proportion of patients with a PSADT risk of good (≥9.00) increased from 59% (*n* = 23) at baseline to 87% (*n* = 34) after 18 months of therapy (Figure 3). The pre-treatment (baseline) distribution of PSADT risk groupings improved after 18 months of P-MCP therapy, with the proportion of patients in the poor risk PSADT group decreasing from 8% to 0%. Similarly, the proportion of patients in the intermediate PSADT risk group decreased from 33% to 13%, and the proportion of patients in the good risk group increased from 59% to 87% (Table 1 and Table 2). There was a significant change in the median PSADT after 18 months of P-MCP therapy (Table 2) as compared to baseline (Table 2) for patients with a pre-treatment PSADT good risk (45.9 versus 14.7 months, *p* = 0.027) and intermediate risk (22.75 versus 5.1 months, *p* = 0.0015). Disease progression during the 18 months of therapy was observed in 18% (*n* = 7) of patients, 8% (*n* = 3) of which had PSA progression only (without radiological progression) and 10% (*n* = 4) had both PSA and radiologic progression.

Toxicity and compliance: None of the patients had grade 3/4 toxicity during the 18 months of therapy. Grade 1 toxicity was observed in 30% (*n* = 12) of patients during the first 6 months of therapy, and in 23% (*n* = 9) during the subsequent 12 months of therapy. This was transient and reversible bloating that did not require treatment discontinuation.

## 4. Discussion

Prostate cancer is a leading cause of death in men worldwide [1], with non-metastatic biochemical disease progression after primary treatment presenting as a therapeutic challenge [10,20]. In the setting of BRPC-M0. there is no proven standard therapy [2,10,11,14,20]. While ADT is effective in lowering PSA level, its long-term survival benefit is not proven, and it is toxic. Thus, there is a need to investigate less toxic compounds in this setting [26,28,30,31,41].

In an earlier report of the present trial, 6-month therapy with PectaSol^®^-Modified Citrus Pectin (P-MCP; EcoNugenics Inc., Santa Rosa, CA, USA) [30] led to a benefit in a significant proportion (78%) of patients with BRPC-M0. Herein, we reported continuation of a durable benefit after 12 more months of therapy in this cohort, with 85% of patients having a durable long-term response, 62% a decreased/stable PSA (versus baseline pre-treatment PSA), 90% a PSADT improvement (versus pre-treatment PSADT), and all with negative scans.

PSADT is the most important clinical prognostic factor in patients with BRCP-M0 and is associated with the development of future metastases [10,13,15,16,17,25]. Lengthening of PSADT may be indicative of an effective therapeutic intervention [52]. Thus, the findings of the present study may suggest that therapy with P-MCP is active in managing BRPC-M0 patients over a durable period. Furthermore, most (95%) of the patients in the present study improved or kept to their PSADT risk grouping. Especially, all patients with a baseline pre-treatment poor PSADT risk, improved their risk grouping. Finally, the benefit of long-term P-MCP therapy was shown without significant toxicity, suggesting that P-MCP can be safely administered [16,20]. A major limitation of the present study is the lack of a placebo (control) arm. Additionally, the present study cohort is relatively small. Furthermore, although retrospective studies have shown that PSADT is a strong predictor of metastasis-free survival, overall survival [11,13], or both [26,53], further validation is required to establish whether change in PSADT is an acceptable endpoint for clinical trials in this patient population. Finally, due to the relatively small cohort of the present study, we were unable to correlate our results with the type of local therapy and to analyze predictive factors for the efficacy of P-MCP therapy (e.g., medications, smoking status, level of androgens, and genetic alterations). Moreover, genetic data were not collected during the present study. These should be tested in future studies. Nonetheless, numerically, P-MCP therapy in the present study was associated with a significant improvement of the expected progression rate in these patients, since, as per the historical data on the natural history of BRPC-M0, without active therapy, 80% of patients are expected to progress within 6 months [30]. In conclusion, in the present study we demonstrated that P-MCP therapy may be associated with a sustained benefit and is safe in patients with BRPC-M0. Future randomized larger clinical trials are needed to validate the present study results and to reveal the underlying mechanism of P-MCP therapy as a galectin inhibitor [38].

## Figures and Tables

**Table 1:** Pre-treatment (baseline) patient characteristics.

Parameter	*n* = 39
Age (years): Median (range)	75 (52–88)
Gleason: % (*n*)	
6	41% (*n* = 16)
7	38% (*n* = 15)
8–10	21% (*n* = 8)
Local therapy: % (*n*)	
Radical prostatectomy	18% (*n* = 7)
Radiation therapy	54% (*n* = 21)
Surgery+RT	28% (*n* = 11)
Prior ADT	38% (*n* = 15)
PSA (ng/mL): Median (range)	4.1 (0.28–30)
PSADT (months) risk grouping: % (*n*)	
Poor < 3	8% (*n* = 3)
Intermediate 3–8.99	33% (*n* = 13)
Good ≥ 9	59% (*n* = 23)
PSADT (months): Median (range)	
Whole cohort	10.3 (1.4–55)
Poor PSADT risk	1.6 (1.4–1.8)
Intermediate risk	5.12 (3.5–8.2)
Good risk	14.74 (9.10–54.6)

**Table 2:** Treatment characteristics and responses after 18 months of PectaSol^®^-Modified Citrus Pectin (P-MCP) therapy.

Parameter	Whole Cohort (*n* = 39)	According to Pre-Study PSADT (Months) Risk Grouping		
		Poor	Intermediate	Good
		<3.00	3.00–8.99	≥9.00
		(*n* = 3)	(*n* = 13)	(*n* = 23)
Overall response to therapy				
(decrease or stabilization of PSA, and/or lengthening of PSADT, with negative scans)	85% (*n* = 33)	66% (*n* = 2)	77% (*n* = 10)	91% (*n* = 21)
PSA response				
Stable/decreased	54% (*n* = 21)	67% (*n* = 2)	23% (*n* = 3)	70% (*n* = 16)
Progression	46% (*n* = 18)	33% (*n* = 1)	77% (*n* = 10)	30% (*n* = 7)
PSADT (months): Median (range)	43.5 (3.5–981)	9.8 (6–200)	18.3 (6.7–500)	47.7 (3.5–981)
PSADT (months) risk grouping: % (*n*)		0% (*n* = 0)	13% (*n* = 5)	87% (*n* = 34
PSADT lengthening	90% (*n* = 35)	100% (*n* = 3)	92% (*n* = 12)	87% (*n* = 20)
Change to a better PSADT risk grouping	36% (*n* = 14)	100% (*n* = 3)	85% (*n* = 11)	not applicable
Radiologic response				
Negative scans	90% (*n* = 35)	67% (*n* = 2)	75% (*n* = 11)	96% (*n* = 22)
Disease progression	10% (*n* = 4)	33% (*n* = 1)	15% (*n* = 2)	4% (*n* = 1)

## Data Availability

The data presented in the study are available with the investigator Daniel Keizman.
